# Signal-mediated localization of *Candida albicans* pheromone response pathway components

**DOI:** 10.1093/g3journal/jkaa033

**Published:** 2020-12-14

**Authors:** Anna Carolina Borges Pereira Costa, Raha Parvizi Omran, Chris Law, Vanessa Dumeaux, Malcolm Whiteway

**Affiliations:** 1 Department of Biology, Concordia University, Montreal, QC H4B 1R6, Canada; 2 Centre for Microscopy and Cellular Imaging, Concordia University, Montreal, QC H4B 1R6, Canada; 3 PERFORM Centre, Concordia University, Montreal, QC H4B 1R6, Canada

**Keywords:** Candida albicans, pheromone response pathway, MAP kinases, mating, opaque cells

## Abstract

A MAPK cascade consists of three kinases, (MEKK, MEK and MAPK), that are sequentially activated in response to a stimulus and serve to transmit signals. In *C. albicans* and in yeast, an MAPK cascade is linked to the pheromone pathway through a scaffold protein (Cst5 and Ste5, respectively). Cst5 is much shorter and lacks key domains compared to Ste5, so in *C. albicans*, other elements, in particular the MEKK Ste11, play key roles in controlling the associations and localizations of network components.

**Abstract:**

*Candida albicans* opaque cells release pheromones to stimulate cells of opposite mating type to activate their pheromone response pathway. Although this fungal pathogen shares orthologous proteins involved in the process with *Saccharomyces cerevisiae*, the pathway in each organism has unique characteristics. We have used GFP-tagged fusion proteins to investigate the localization of the scaffold protein Cst5, as well as the MAP kinases Cek1 and Cek2, during pheromone response in *C. albicans*. In wild-type cells, pheromone treatment directed Cst5-GFP to surface puncta concentrated at the tips of mating projections. These puncta failed to form in cells defective in either the Gα or β subunits. However, they still formed in response to pheromone in cells missing Ste11, but with the puncta distributed around the cell periphery in the absence of mating projections. These puncta were absent from *hst7Δ/Δ* cells, but could be detected in the *ste11Δ/Δ hst7Δ/Δ* double mutant. Cek2-GFP showed a strong nuclear localization late in the response, consistent with a role in adaptation, while Cek1-GFP showed a weaker, but early increase in nuclear localization after pheromone treatment. Activation loop phosphorylation of both Cek1 and Cek2 required the presence of Ste11. In contrast to Cek2-GFP, which showed no localization signal in *ste11Δ/Δ* cells, Cek1-GFP showed enhanced nuclear localization that was pheromone independent in the *ste11Δ/Δ* mutant. The results are consistent with CaSte11 facilitating Hst7-mediated MAP kinase phosphorylation and also playing a potentially critical role in both MAP kinase and Cst5 scaffold localization.

## Introduction

*Candida albicans* is an ascomycete yeast closely related to *Saccharomyces cerevisiae*; recent estimates suggest a divergence time of 235 Mya (Taylor and Berbee 2006). Because it is such a well-studied model organism, *S. cerevisiae* is frequently used to direct understanding of biological processes in *C. albicans*. Although common cellular processes are typically directed by orthologous pathways in the two fungi, these pathways can exhibit diversified connectivity and outputs that can contribute to adaptation to either the non-pathogenic or the commensal/pathogenic lifestyles of the two organisms.

In an apparent fundamental divergence from *S. cerevisiae*, *C. albicans* shows no evidence for undergoing conventional meiosis, and sporulation has never been observed (Bennett 2015; Noble *et al.* 2017). Instead this fungus can reproduce through a parasexual mechanism of concerted chromosome loss in which tetraploid cells formed by mating of diploid cells return, after nuclear fusion, to a diploid or near diploid state (Bennett and Johnson 2003, 2005; Bennett 2015). As well, while the haploid cells of *S. cerevisiae* are constitutively competent for mating, the typically diploid *C. albicans* must undergo a phenotypic transition prior to mating; it must switch from the standard white yeast morphology to the elongated opaque form to become mating competent. This transition occurs in rare cells homozygous for the *MTL-*locus, (*MTL* a/a or α/α), and is tightly controlled by the transcription factor Wor1 and additional regulators under opaque inducing conditions (N-acetylglucosamine, ≥5% CO_2_ and acidic pH) (Huang *et al.* 2006; Zordan *et al.* 2007; Huang *et al.* 2009, 2010; Sun *et al.* 2015).

The mating process in both *S. cerevisiae* and in *C. albicans* opaque cells initiates with secretion of pheromones to stimulate cells of opposite mating type to induce polarized growth toward the stimulus (Bennett and Johnson 2003, 2005; Merlini *et al.* 2013). The *C. albicans* sexual projections can be highly elongated, and they resemble a filamentous form more than they do the shmoos formed by *S. cerevisiae* (Lockhart *et al.* 2002, 2003; Bennett and Johnson 2003; Soll 2014). The a and α pheromones are sensed by the respective pheromone receptors Ste3 and Ste2 on the surface of the cell, and this receptor-ligand binding in turn activates a heterotrimeric G protein. The dissociation of the Gα subunit releases the Gβγ subunits that are predicted to remain at the plasma membrane. In the baker’s yeast, the Gβ subunit (Ste4) recruits the scaffold protein Ste5 to the plasma membrane. Subsequently, this membrane recruitment of Ste5, itself complexed with the mitogen-activated protein kinase cascade (MAPK cascade) proteins, namely the MAP/ERK kinase kinase (MEKK) Ste11, the MAP/ERK kinase (MEK) Ste7, and the MAP kinase (MAPK) Fus3, brings the MAPK cascade kinases close to the protein kinase Ste20 which is tethered at the membrane through its interaction with Cdc42. This makes Ste11 available for phosphorylation and activation by Ste20. This activation triggers the cascade, ultimately turning on the downstream components of the MAPK pathway and leading to activation of the transcription factor Ste12 responsible for the induction of the pheromone responsive-genes (Whiteway *et al.* 1995; Elion 2001; Bardwell 2005; Takahashi and Pryciak 2008; Malleshaiah *et al.* 2010; Merlini *et al.* 2013). In *S. cerevisiae*, the pheromone stimulation changes the expression of about 3% of the genome, arrests the cells in the G1 phase of cell-cycle and orients shmoo formation toward the mating partner, thus facilitating mating (Bardwell 2005).

The yeast scaffold protein Ste5 provides specificity to the pheromone response pathway as it is required for insulation of the MAPK components from participation in other pathways, and is required for efficient activation of Fus3, the major MAPK specific for this pathway (Elion 2001; Good *et al.* 2009). The N-terminal half of the Ste5 contains two domains, the RING (RING-H2 Zn finger domain) and PH (plextrin homology) domains, implicated in protein-protein interactions for dimerization with another Ste5 molecule, for binding the protein kinases Ste11 and Fus3, and for association with the Gβ subunit Ste4 (Kranz *et al.* 1994; Choi *et al.* 1994; Whiteway *et al.* 1995; Yablonski *et al.* 1996). The C-terminal half the scaffold protein harbors a vWA (von Willebrand Type A) module responsible for interaction with Ste7 (Good *et al.* 2009). However, Ste5 is not simply a passive scaffold. Ste5 plays a direct role in the activation of the MEKK Ste11 by localizing it to the membrane-associated Ste20 and by mediating allosteric control through binding to the N-terminal regulatory domain of Ste11 (Choi *et al.* 1994; Marcus *et al.* 1994). Furthermore, the vWA domain of Ste5 associates with Ste7 to form a catalytically competent complex that makes the Fus3 activation loop accessible to Ste7 (Good *et al.* 2009).

The pheromone response pathway in *C. albicans* is mediated by orthologous proteins to those of *S. cerevisiae* and the input and output of the pathways are similar, but the pathway circuit details in *C. albicans* are distinct. Côte *et al.* (2011) and Yi *et al.* (2011) simultaneously characterized the MAPK scaffold protein Cst5 in *C. albicans*. They observed that although Cst5 is approximately half the size of its orthologue Ste5 due to the lack of the catalytic vWA domain, the scaffold is still relevant for tethering the protein kinases of the mating MAPK cascade, and fully required for mating. Furthermore, both the *C. albicans* Gα and Gβ subunits are required for mating, for morphological changes and for transcription induction, indicating that both subunits are positively involved in the transmission of the mating signal, whereas in *S. cerevisiae* the Gα subunit is a negative regulator of the pheromone pathway (Dietzel and Kurjan 1987; Dignard *et al.* 2008).

In this study, we investigated the relationships among the pheromone components to identify how the MAPK proteins are coordinated through a scaffold that lacks a key domain. An attempt to bypass other components by artificially driving either Cst5 or Ste11 to the plasma membrane suggested that the interactions among the proteins are more complex than the linear cascade characterized in *S. cerevisiae*. Indeed, it appears that Ste11 interacts with Cst5 to generate a complex node to tether the downstream kinases where Ste11 can both act enzymatically on Hst7 as well as make the MAPK Cek1 available for phosphorylation and activation by Hst7.

## Materials and methods

### Strains, media and growth conditions

All strains used in this study are listed in Supplementary Table S1. *C. albicans* strains were routinely cultivated on YPD agar (1% yeast extract, 2% bacto-peptone, 2% D-glucose, 2% agar and 50 µg/mL uridine) at 30^°^/48 h. Switching from the white to the opaque form was induced at 25^°^ for 5–7 days on synthetic complete agar supplemented with 1.25% N-acetylglucosamine (GlcNAc), 100 µg/mL uridine and containing 5 µg/mL phloxine B to stain opaque colonies.

### Strain construction

The transient CRISPR-Cas9 system method was used to make deletions and insertions at both alleles (Min *et al.* 2016). The list of primers and plasmids are shown in Supplementary Table S2. The strain SN148 (*arg4/arg4 leu2/leu2 his1/his1 ura3/ura3*) homozygous for the *MTL* locus a/a was used as a wild-type and background strain for the transformations. The plasmid pV1093 was used to construct the Cas9 cassette and sgRNA expression cassette. The optimal guide RNAs for the constructs were designed on Benchling (Vyas *et al.* 2015). The plasmid pFA-CaARG4 was used as a template to amplify the repair DNAs to delete *CAG1*, *STE2*, *STE4*, *STE11*, *HST7* and *CST5*. The 3ʹ end of the ORFs of *STE11* (73 nucleotides), *HST7* (55 nucleotides) and *CST5* (17 nucleotides) were conserved to preserve the promoter site and termination site of a closely adjacent gene and an overlapping gene, respectively. The *ste11Δ/Δ CST5-GFP* mutant was used as a background strain to construct the double mutants with *CAG1*, *STE4* or *HST7* deletion using the plasmid pFA-CmLEU2 as a template for the repair DNAs.

The scaffold protein *CST5* was tagged with GFP at the 3ʹ end using the transient CRISPR-Cas9 system method (Min *et al.* 2016). The Cas9 cassette and sgRNA expression cassette were constructed as mentioned above. Since the 3ʹ end of *CST5* overlaps 17 nucleotides with the 3ʹ end of *SPO71*, the repair DNA to tag *CST5* with GFP-HIS was synthesized in steps, not to disrupt the 3ʹ end of *SPO71* and alter the PAM sequence. Plasmid pFA-GFP-CaHIS1 was used to amplify the GFP gene and the selectable marker. To move *SPO71* beside the GFP-HIS1 tagging and alter the PAM sequence, two overlapping fragments of DNA were amplified from SC5314 *C. albicans* genomic DNA so that the reverse primer (P2_PAM19.2129) of the first fragment and the forward primer (P3_PAM19.2129) of the second fragment overlapped and carried a point mutation to introduce a silent mutation (exchange of G by A at the position 2345). These two fragments were joined by single-joint PCR reaction (Yu *et al.* 2004) followed by a third round of PCR reaction with nested primers (P3_orf19.2129/P6_PAM19.2129). Next, GFP-HIS1 and altered 3ʹ end of *SPO71* fragments were joined by single-joint PCR reaction (Yu *et al.* 2004), amplified with nested primers (P5_GFP_HIS1/S2-orf19.2129) and gel extracted (Zymoclean Gel DNA recovery kit) to be cloned into the pCR2.1 TOPO vector (Invitrogen). Five microliters of the ligation product was transformed into *Escherichia coli* DH5α and positive clones were selected on LB agar (1% bacto-tryptone, 0.5% yeast extract, 1% NaCl, 2% agar) supplemented with 50 µg/mL ampicillin. The plasmids carrying the insert were isolated using the miniprep (Bio Basic) kit and confirmed by digestions with *SalI* and *HindIII*-HF and sequencing.

*CST5* was also tagged at the 3ʹ end with the amino acid sequence CCTIV, the CAAX sequence from CaSte18. For this, the sgRNA expressed cassette designed to tag *CST5* with GFP was used to guide the Cas9 cassette and the plasmid TOPO vector carrying the insert GFP-HIS1+ altered 3ʹ end of *SPO71* was used as a template to tag *CST5* with CAAX-HIS1 using the long primers CST5-CAAX-F1b/CST5-R1.

The 3ʹ end of *STE11* was tagged with amino acid sequence CCVIV, the CAAX sequence from CaRas1, using the plasmid pFA-CaHIS1 as template to amplify the repair DNA. To insert the tail and selectable marker by CRISPR-Cas9 method, 210 nucleotides were deleted downstream the stop codon to tag *STE11* with the CAAX sequence.

The MAPKs *CEK1* and *CEK2* were also tagged with GFP at the 3ʹ end using as a template to amplify the repair DNAs using the plasmid pFA-GFP-CaHIS1. For *CEK1*, 133 nucleotides, and *CEK2*, 79 nucleotides, downstream of the genes were deleted to make the insertions using the CRISPR-Cas9 system.

The PCR products were purified and concentrated by ethanol precipitation and transformed into *C. albicans* at concentrations of 3 µg of repair DNA, 1 µg of sgRNA expression cassette and 1 µg of CaCas9 cassette using the lithium acetate transformation method (Walther and Wendland 2003). The positive clones were selected on SD agar (2% glucose, 0.17% yeast nitrogen base with ammonium sulfate, 2% agar) supplemented with the appropriated amino acid when needed. The resultant colonies were genotyped by colony PCR to confirm the right integration.

### Fluorescence microscopy

Opaque cells were cultivated in liquid SC-GlcNac at room temperature for 24 h with shaking at 220 rpm. Next, the culture was adjusted to OD_600_ at 0.8 in 2 tubes of 10 mL liquid SC-GlcNac medium in which one tube was treated with 10 µg/mL α-pheromone and the other tube was untreated. After 6 h of incubation at room temperature with shaking, the cells were collected and washed twice with sterile MilliQ water. When required, the cells were stained with 2 µg/mL of DAPI (Invitrogen) for 5 min or 10 µg/mL of Hoechst 33342 (Invitrogen) for 20 min in the dark. The GFP strains and strains stained with the fluorescent dyes were prepared for live yeast cell imaging using a Leica DM6000B epifluorescence microscope (Leica Biosystems) with Volocity acquisition software (PerkinElmer) using a 100× Leica Plan Fluotar (NA 1.3) lens, an Orca ER camera (Hamamatsu), and excitation/emission filters for UV (377/50ex, 447/60em) and GFP (480/20ex, 510/20em). We used the same excitation intensity and exposure time to capture the images and the representative pictures shown in the figures were adjusted uniformly.

### GFP signal quantification

The live-cell imaging was captured and analyzed by FIJI (Schindelin *et al.* 2012). To quantify the fluorescent intensity (arbitrary units), the opaque cells were outlined using the elliptical selections tool to measure the “mean gray value” in the DIC channel and then, quantified in the FITC channel. The “mean gray value” of the background for each picture was subtracted from the “mean gray value” of the cells circled in the same picture.

Intensity of puncta or nuclear signals was quantified using FIJI (Schindelin *et al.* 2012) scripts. Cells were first outlined using DIC images passed to the Yeastspotter Convolutional Neural Net (He *et al.* 2020; Lu *et al.* 2019); well-defined cells were selected based on a DIC image by the observer, and then processed as follows. To analyze nuclear signals, well-defined cells were selected in the DIC channel (by an observer blind to the GFP distribution), then the Hoechst channel was treated with a Gaussian blur and thresholded according to Otsu's criteria to form a binary mask of nuclei; small (<200px) objects were removed from consideration. Each cell outlined by the CNN and selected by the observer was then quantified by measuring the integrated GFP intensity and the area of both the cell and nucleus; the mean intensity of each compartment was derived from these measurements (integrated intensity/pixel count = mean intensity). To analyze the intensity of GFP puncta, cells of interest were selected as above, and the area and mean intensity of the GFP channel were calculated; the “Find Maxima” command was used on the GFP channel within individual cells, a 5 × 5 pixel square was drawn around each maxima found, and the mean intensity of all of these squares is reported on a cell-by cell basis as the “Mean Puncta Intensity.”

### Mating assay

Opaque cells of strains 3315α and 3745a were used as tester strains. They have the auxotrophic markers *trp1/trp1* and *lys2/lys2*. Opaque cells of SN148a and mutant strains were streaked out as straight lines on SC-GlcNac+Phloxine B, as well as the tester strains on another plate. After 48 h of incubation at 25^°^, the experimental plate and tester plates were replica plated together onto SC-GlcNac+Phloxine B and YPD and allowed to mate at 25^°^/24 h. Subsequently, the mating plate was replicated on SD agar lacking tryptophan, lysine, uridine and leucine at 30^°^ for 3 days for prototrophic selection (Dignard *et al.* 2008). Next, the plates were scanned.

### Western blotting

Opaque cells were cultivated in liquid SC-GlcNac medium and treated with α-pheromone as described above. The cells were harvested by centrifugation, washed with sterile MilliQ water, frozen at −80^°^ and lyophilized. Lyophilized cells were resuspended in HK buffer (Liu *et al.* 2010) and lysed by vortexing (30 seconds vortexing followed by 3 min on ice, 10 cycles). Extracted proteins were quantified by Bradford assay (Bio-Rad). Briefly, 30 µg of proteins were boiled with SDS gel loading dye and resolved in 4–12% gradient SDS polyacrylamide gels. Proteins were transferred to nitrocellulose membrane (Bio-Rad). Next, membrane was blocked with Tris-buffered saline Tween [TBST; Tris (pH 7.5), 137 mM NaCl, 0.1% Tween 20] containing 5% skim milk for 1 h, then rinsed 3× with TBST for 15 min and blotted with anti phospho-p44/42 MAPK antibody (Cell Signalling, 9101) diluted 1:1000 at 4^°^/overnight. Blots were washed 3× with TBST, blotted with 1:10,000 dilution of anti-rabbit IgG-HRP (Santa Cruz Biotechnology, sc-2357) for 1 h and washed 3× with TBST. Blots were developed using the Amersham ECL Western blotting analysis system. Blots were stripped and blotted with anti-α-tubulin antibody (Sigma-Aldrich, T5168) diluted to 1:1000 for 2 h as a loading control. After washing, membrane was blotted with 1:10,000 dilution of anti-mouse m-IgGk BP-HRP antibody (Santa Cruz Biotechnology, sc-516102) for 1 h followed by developing.

### RNA-seq analysis

The wild-type strain (SN148a) untreated and treated with α-pheromone for 6 h, and untreated *ste11Δ/Δ* as described above were subjected to RNA extraction in two biological replicates using the QIAGEN RNA extraction kit protocol. The cells were disrupted completely with bead beater shaking 25 times for 20 s with 1 min on ice between treatments (Rastghalam *et al.* 2019). Samples were tested for quality control using Bioanalyzer and submitted for sequencing to the Genome Quebec Innovation Centre using Illumina miSeq sequencing platform. RNA-seq data was processed and analyzed as described by Costa *et al.* (2019). Raw and processed data have been deposited in NCBI’s Gene Expression Ominibus and are accessible through GEO Series accession number GSE158062 (https://www.ncbi.nlm.nih.gov/geo/query/acc.cgi? acc=GSE158062) (Edgar *et al.* 2002).

### Statistical analysis

The results were analyzed by Kruskal–Wallis test, Dunn’s multiple comparisons test and Mann-Whitney test as indicated in the text. GraphPad Prism version 6.00 was used for the analysis of results.

### Data availability and deposited data

Strains and plasmids are available upon request. Supplementary files are available at FigShare. Strains used in this study are listed in Supplementary Table S1. The list of primers and plasmids are in Supplementary Table S2. Supplementary material presents the RNA-sequencing data. RNA-sequencing data are also accessible through GEO Series accession number: GSE158062. 

Supplementary material is available at figshare: https://doi.org/10.25387/g3.13013894.

## Results

### The deletion of the G proteins Cag1 and Ste4 and protein kinases Ste11 and Hst7 perturb the mobilization of the scaffold protein Cst5 in distinct ways

**Figure 1:**
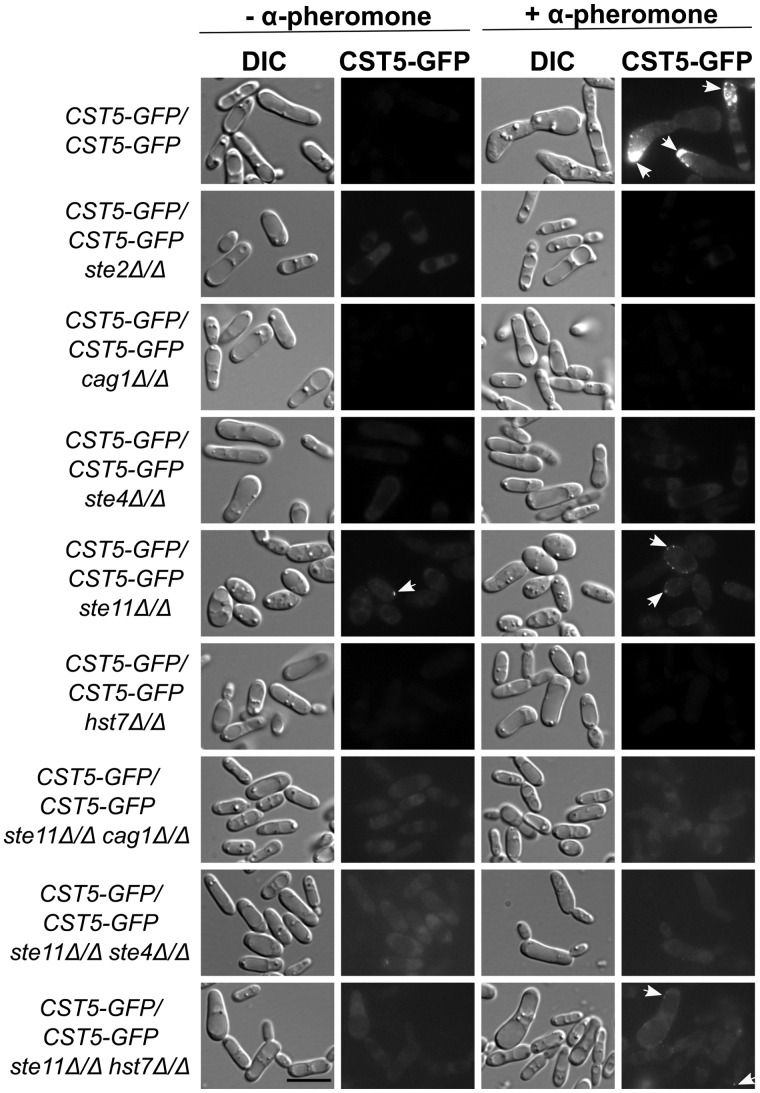
Cst5-GFP localization under pheromone stimulation in the absence of *STE2, CAG1*, *STE4*, *STE11*, and *HST7*. Opaque cells were not treated (−) or treated (+) with 10 µg/mL α-pheromone for 6 h at room temperature and examined under the microscope. The localization of puncta is indicated by white arrows. Representative pictures are shown for cells imaged under differential interference contrast (DIC) and GFP channels. Scale bar: 10 µm.

A challenge in establishing the relationships of elements in fungal pheromone pathways is that null mutants of the pathway members typically provide identical phenotypes - specifically defects in each of mating ability, mating projection formation and transcription induction (Stevenson *et al.* 1992; Bardwell 2005). To enhance phenotypic analyses in the *C. albicans* pathway, we tagged the scaffold protein Cst5 with GFP and monitored its intracellular location in response to pheromone in various mutant backgrounds. Live-cell imaging revealed that none of the strains mutant in the genes *STE2*, *STE4*, *CAG1*, *HST7* or *STE11* formed sexual projections under pheromone stimulation, unlike the wild-type strain (SN148a) (Figure 1). When treated with pheromone, wild-type cells containing Cst5-GFP responded to pheromone stimulation by localizing the GFP signal in punctate structures at the tips of the sexual projections, a behavior similar to that shown by Ste5 in *S. cerevisiae* (Pryciak and Huntress 1998). By contrast, the strains mutant for the genes *CAG1* and *HST7* showed no tagged protein signal with or without pheromone stimulation, while the strains lacking *STE2* and *STE4* expressed significant cytoplasmically-distributed Cst5-GFP signal in the absence of pheromone. When treated with pheromone, the *ste4Δ/Δ* mutant showed an increase in this background signal to a level comparable to that of the pheromone-treated wild-type, while the *ste2Δ/Δ* strain showed no increase. Dramatically, the *ste11Δ/Δ* mutant not only expressed the Cst5-GFP signal at high levels without stimulation, but when exposed to pheromone also exhibited a clear signal concentrated in puncta (Figure 1). These puncta were similar to the puncta which formed in the pheromone-treated wild-type cells and were broadly distributed at the edges of the cells, even in the absence of mating projections (Figure 1).

The observation that Cst5-GFP formed pheromone-stimulated cell surface puncta in the *ste11Δ/Δ* mutant is consistent with observations in *S. cerevisiae*, as the Ste5 scaffold formed pheromone-mediated membrane complexes that were dependent on the G protein subunits, but were independent of the downstream kinases (Pryciak and Huntress 1998). The absence of a GFP signal in the Hst7 mutant strain was inconsistent with the observations in *S. cerevisiae*, because Hst7 functions downstream of Ste11 in the kinase cascade. Therefore, we constructed double mutants of *STE11* in combination with *CAG1*, *STE4* or *HST7*. The double mutants all presented in some level of Cst5-GFP background, and the *ste11Δ/Δ hst7Δ/Δ* strain also showed some signal concentrated in cell periphery-associated puncta in the pheromone treated cells (Figure 1). The dramatic appearance of the Cst5-GFP signal in mutant backgrounds such as *ste11Δ/Δ* occurs in the absence of any enhanced expression of *CST5* in these cells. An interesting possibility is that the GFP signal is appearing due to a change in the context of the protein associations or cellular localization of Cst5 that allows GFP signalling in the absence of Ste11, as well as Ste2 and Ste4. In any event, the appearance of the signal allows us to probe the cellular location and context of the protein in those cells generating a visible signal.

**Figure 2:**
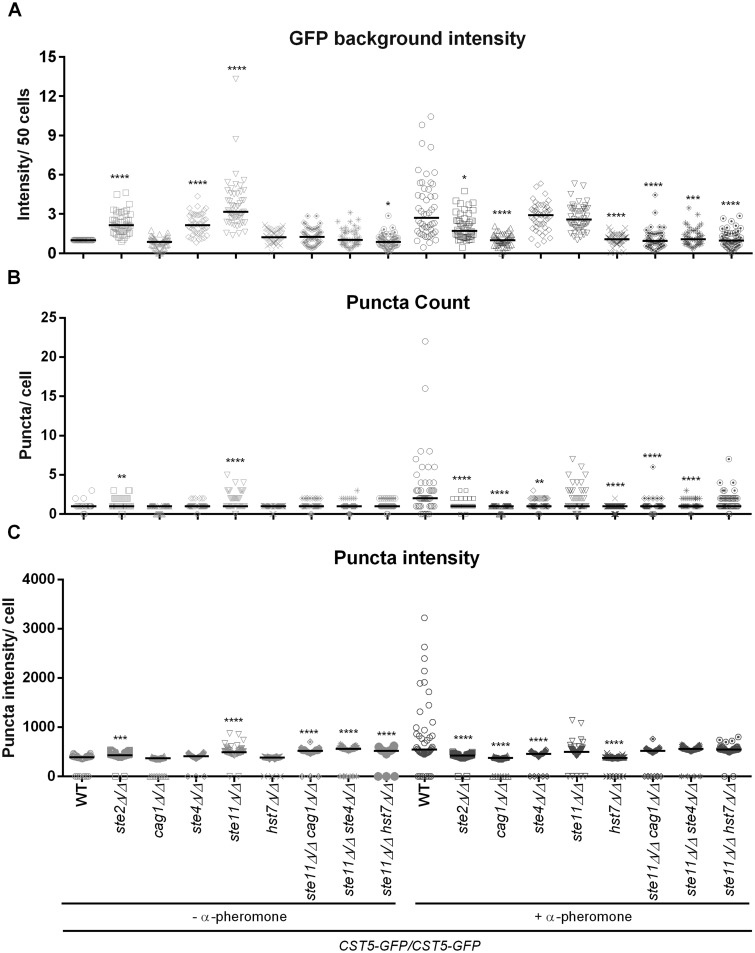
Cst5-GFP signal quantification. Opaque cells were not treated (−) or treated (+) with 10 µg/mL α-pheromone for 6 h at room temperature, examined under the microscope and the images captured were analyzed by FIJI to quantify Cst5-GFP background signal (A), puncta count (B) and puncta intensity (C). Fifty cells were analyzed for each strain/condition from two biological replicates. The values of GFP background intensity (A) were normalized to the non-treated *CST5*-GFP/*CST5*-GFP (WT) average value. Horizontal lines show the median. *P <* *0.05, **P <* *0.01, ***P <* *0.001, ****P <* *0.0001, strains that differ from the wild-type in the same condition (Kruskal–Wallis test, Dunn’s multiple comparison test).

The behavior of the Cst5-GFP signal, in terms of the general background and concentration as puncta, was quantified using FIJI revealing a range of signal intensities within the same strain and condition (Figure 2A). In the absence of pheromone stimulation, the mutants *ste2Δ/Δ*, *ste4Δ/Δ*, *ste11Δ/Δ* and *ste11Δ/Δ hst7Δ/Δ* presented significant Cst5-GFP background when compared with the wild-type strain (Figures 1 and 2A), while the *hst7Δ/Δ* and *cag1Δ/Δ* strains showed no signal. The wild-type strain readily responded to pheromone stimulation with a higher background Cst5-GFP signal, for which the *ste4Δ/Δ* and *ste11Δ/Δ* mutants showed statistically similar intensities, and formed puncta of intense staining at the sites of mating projections (Figure 2A). The *ste11Δ/Δ* mutant not only expressed a strong Cst5-GFP signal independent of stimulation, but also exhibited signal in puncta in both conditions (Figure 2B), though the large, easily visible puncta similar to those of pheromone-treated wild-type cells appeared only during pheromone stimulation (Figure 1). In the absence of the receptor Ste2, the program detected puncta formation independent of pheromone treatment that was both statistically significant in comparison with the non-responsive wild-type, and statistically different in relation to the stimulated wild-type. Furthermore, the mutants *ste2Δ/Δ*, *ste11Δ/Δ* and the double mutants of *ste11Δ/Δ* in combination with a *CAG1*, *STE4* or *HST7* deletion, displayed detectable puncta intensity compared with the wild-type without pheromone treatment, whereas *ste11Δ/Δ* and the double mutants showed no statistical difference in comparison with the highly responsive wild-type strain when exposed to pheromone (Figure 2C). The regulation of the scaffold protein localization seems to be dependent on physical interactions with upstream components such as the G protein subunits, as well as downstream components such as the MEK Hst7, under the no pheromone “standby” mode as well as in response to pheromone.

### Cst5-CAAX and Ste11-CAAX constructs did not suppress the deletion of key elements of the pheromone response pathway

**Figure 3:**
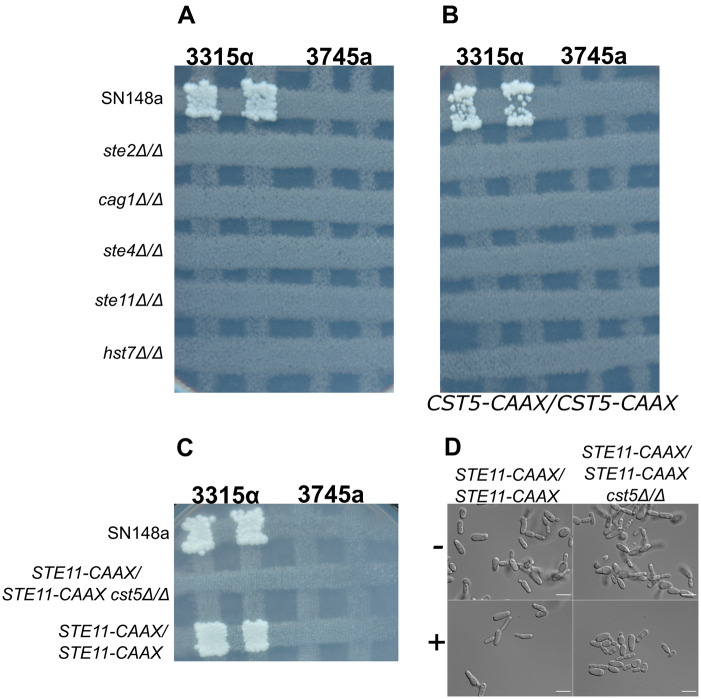
Artificially driving Cst5 and Ste11 to the plasma membrane did not suppress the absence of pheromone components in the mating process. The mutant strains for the genes *STE2, CAG1, STE4, STE11* and *HST7* non-tagged (A) or *Cst5*-CAAX tagged (B) and *STE11*-CAAX/*STE11*-CAAX with or without *CST5* deletion (C) were assayed for mating capacity. The strains 3315α and 3745a were used as mating type testers. They have auxotrophic markers *trp1/trp1* and *lys2/lys2*. Cells from opaque colonies for the tester strains were crossed with SN148a (wild-type) and mutant strains (*MTL*a/a), in the opaque state, on SC- GlcNac and YPD at 25^°^/24 h and then replicated on selective medium SC- glucose (Trp- Lys- Ura- Leu-) at 30^°^ for 3 days to detect auxotrophic mating products. *CST5*-CAAX sequence from CaSte18: Cys-Cys-Thr-Ile-Val. *STE11*-CAAX sequence from CaRas1: Cys-Cys-Val-Ile-Val. (D) Representative pictures of opaque cells of *STE11*-CAAX/*STE11*-CAAX with or without *CST5* deletion non-treated (−) treated (+) with 10 µg/mL α-pheromone for 6 h at room temperature. Scale bar: 10 µm.

To further investigate the relationships among the pheromone players in the mating process, the scaffold protein Cst5 and the MEKK Ste11 were artificially targeted to the plasma membrane to investigate activation of the MAPK cascade in the absence of various pheromone pathway components. Opaque cells of *ste2Δ/Δ*, *cag1Δ/Δ*, *ste4Δ/Δ*, *ste11Δ/Δ* and *hst7Δ/Δ* mutant strains were defective for mating ability (Figure 3A), so we asked whether any of these mutants could be suppressed by the membrane targeted constructs. As a critical element of the pheromone pathway, the *C. albicans* scaffold protein *CST5* was tagged with CAAX motif sequence of CaSte18 (CCTIV); the equivalent modification of *S. cerevisiae* Ste5 targets the scaffold protein to the plasma membrane and triggers pheromone response leading to mating (Pryciak and Huntress 1998). The carboxy-terminal CAAX motif (where C = cysteine, A = aliphatic amino acid, and X = terminal amino acid) undergoes posttranslational modifications in which the AAX motif is cleaved off and a hydrophobic prenyl group is added allowing the protein to bind to the membrane (Cox and Der 1992; Whiteway and Thomas 1994). As shown in Figure 3B, the Cst5-CAAX box construct did not bypass the absence of any pheromone pathway gene inactivation to allow mating.

The MEKK Ste11 was also tagged with the CAAX motif sequence of CaRas1 (CCVIV) to direct anchoring to the plasma membrane and potential activation by the protein kinase Cst20 (Piispanen *et al.* 2011; Gentry *et al.* 2015). The tagged strain effectively mated and responded to pheromone treatment by forming shmoos, showing the CAAX sequence which did not interfere with function, but introducing the membrane-targeted Ste11 construct did not bypass the requirement of the scaffold protein Cst5 (Figure 3, C and D).

### Ste11 plays a role in localizing the MAPK Cek1

**Figure 4:**
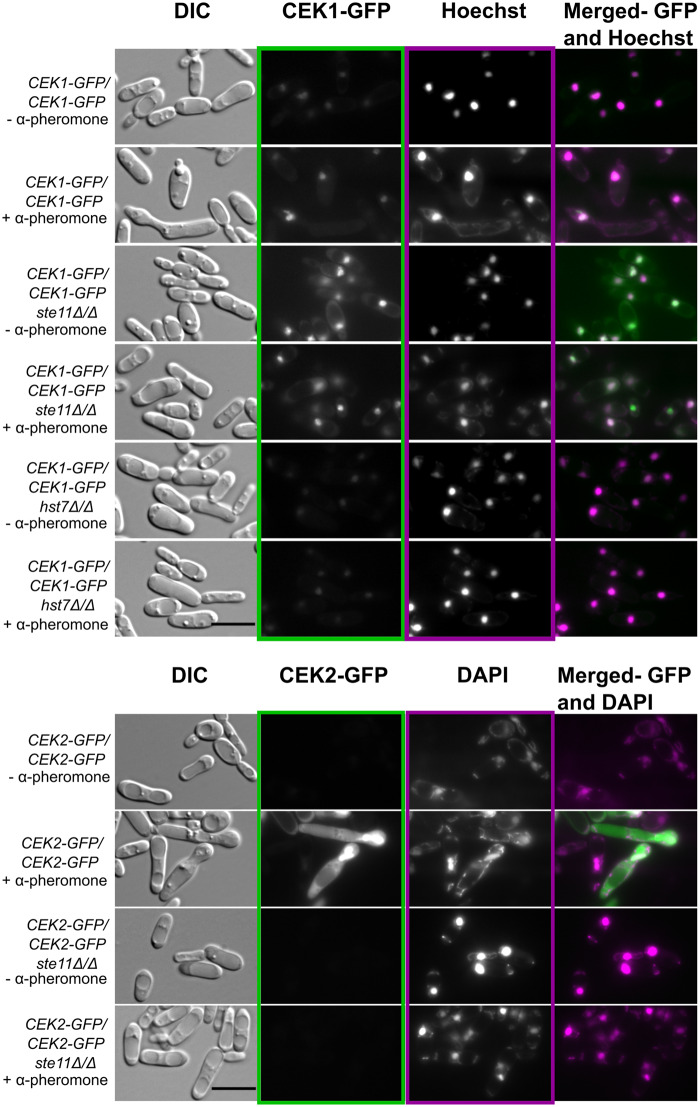
Cek1-GFP signal was highly expressed in *ste11Δ/Δ*, while Cek2-GFP was not detected. Opaque cells were not treated (−) or treated (+) with 10 µg/mL α-pheromone for 6 h at room temperature and examined under the microscope. Representative pictures are shown for cells imaged under DIC and GFP channels and DAPI channel to detect Hoechst or DAPI staining. Merged pictures: GFP channel (Green)/DAPI channel (Magenta). Scale bar: 10 µm.

A *ste11Δ/Δ* opaque cell is defective for shmoo formation and mating but is still able to localize the scaffold protein Cst5-GFP to cell surface puncta. Therefore, we asked whether the pheromone response pathway was truly off in the absence of *STE11*. Under pheromone stimulation the MAPK cascade is activated, and the MAPK Cek1 is phosphorylated at the activation loop while in the cytoplasm after which it is proposed to translocate to the nucleus to phosphorylate and activate the transcription factor Cph1, leading to the expression of pheromone-responsive genes (Bennett and Johnson 2005; Rastghalam *et al.* 2019). To follow this behavior, we tagged the MAPK Cek1 with GFP and monitored it under the microscope. The wild-type strain carrying Cek1 tagged with GFP showed a Cek1-GFP background signal in untreated cells, and responded to pheromone stimulation by increasing the GFP signal in the nucleus (Figure 4) as noted previously (Rastghalam *et al.* 2019). Intriguingly, the *ste11Δ/Δ* mutant exhibited a strong Cek1-GFP signal and nuclear localization independent of pheromone stimulation, while the *hst7Δ/Δ* mutant did not respond to the pheromone treatment and showed a Cek1-GFP signal comparable to that of the non-treated wild-type opaque cells (Figure 4).

The mating pathway in *C. albicans* is also mediated by the MAPK Cek2 which has been proposed to function in the adaptation process (Rastghalam *et al.* 2019). We examined the influence of *STE11* on the behavior of Cek2. In the wild-type strain the Cek2-GFP fusion protein was distributed throughout the cytoplasm and showed clear nuclear localization when treated with pheromone (Figure 4), but this appearance in the nucleus was late in the process, consistent with a role in adaptation. By contrast, Cek2-GFP was not detected in either untreated or treated *ste11Δ/Δ* cells (Figure 4).

**Figure 5:**
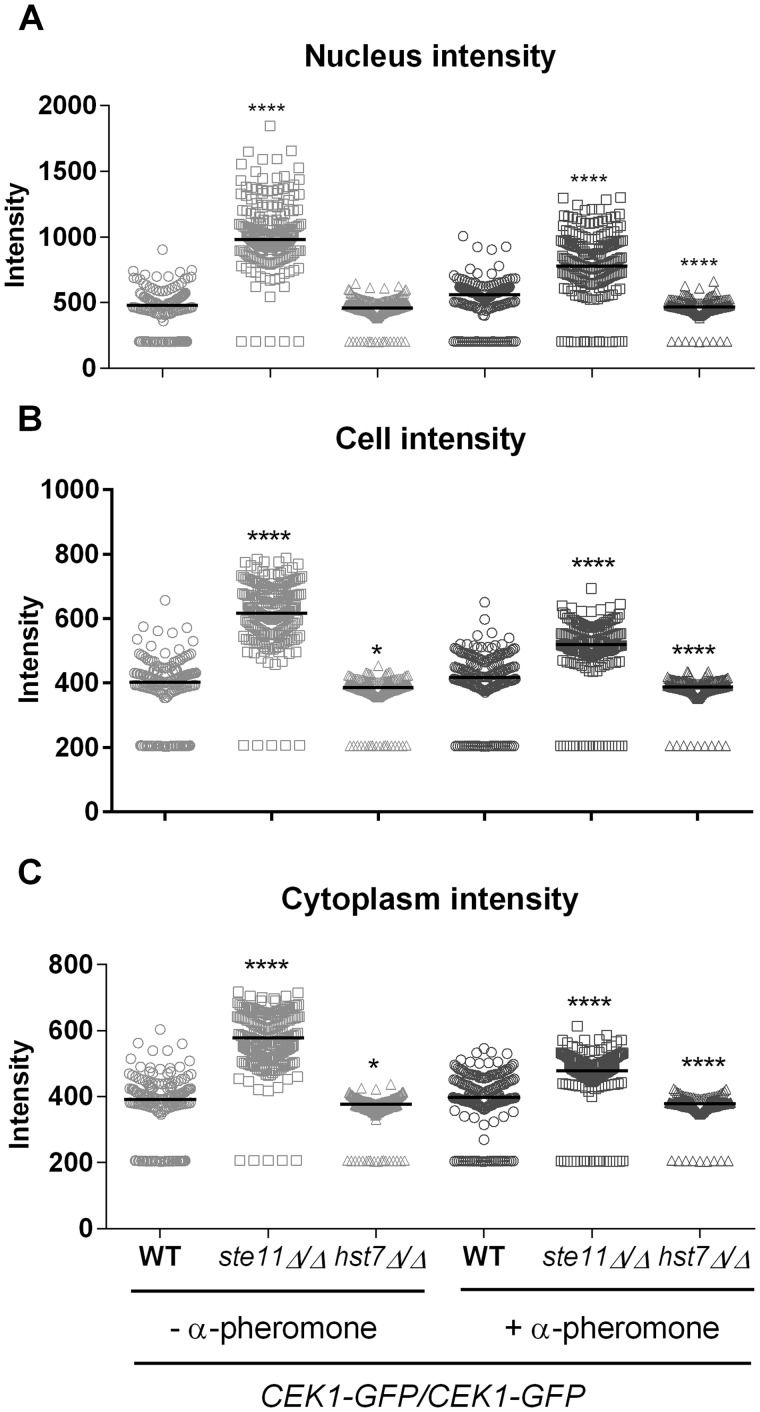
Deletion of *STE11* enhanced Cek1-GFP background signal and nuclear localization independent of pheromone exposure. Opaque cells were not treated (−) or treated (+) with 10 µg/mL α-pheromone for 6 h at room temperature, examined under the microscope and the images captured were analyzed by FIJI. The Cek1-GFP signal intensity was quantified into the nucleus (A) and in the whole cell (B). (C) The cytoplasmic intensity (CI) was calculated using the parameters generated by FIJI using the formula CI= (Cell Integrated Density-Nuclear Integrated Density)/(Cell Area-Nuclear Area). Two hundred cells were analyzed for each strain/condition from two biological replicates. Horizontal lines show the median. *P <* *0.05 and ****P <* *0.0001, strains that differ from the wild-type in the same condition (Kruskal–Wallis test, Dunn’s multiple comparison test).

We quantified the Cek1-GFP signal in the whole cell, in the nucleus, and in the cytoplasm using FIJI. As noted in the images, the wild-type strain (*CEK1*-GFP/*CEK1*-GFP) responded to pheromone treatment by significantly increasing the Cek1-GFP signal in the whole cell, nucleus and cytoplasm when compared with the non-treated wild-type strain (Mann-Whitney test, *P-*values of 0.0005, <0.0001 and 0.0195, respectively) (Figure 5, A–C). The Cek1-GFP signal remained unchanged under all conditions tested in the *hst7Δ/Δ* mutant, and showed statistical differences with the wild-type intensity quantifications (Figure 5, A–C ). Confirming the cell images, deletion of *STE11* substantially enhanced the Cek1-GFP signal in the whole cell (nucleus and cytoplasm) independent of pheromone treatment. It is interesting to note that under pheromone stimulation the Cek1-GFP signal intensities in the cytoplasm and nucleus in the *ste11Δ/Δ* mutant were slightly lower compared with those of the untreated *ste11Δ/Δ* mutant but the intensities were still statistically higher than those presented by the treated wild-type (Figure 5, A–C ). The lack of *STE11* interferes with Cek1 regulation but under pheromone treatment the Cek1 levels tend to decrease perhaps due to the influence of other MAPK components, which, after activation, may form an incomplete complex allowing moderate Cek1 regulation.

**Figure 6:**
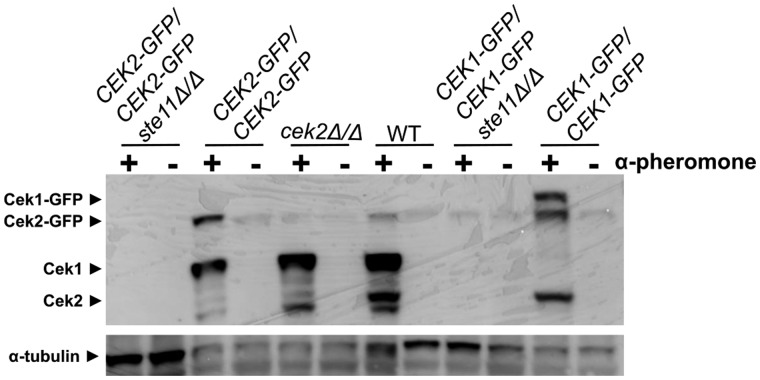
Ste11 is required for transferring the signal leading to phosphorylation of both MAP kinases Cek1 and Cek2. Western blot of proteins extracted from opaque cells non-treated (−) or treated (+) with 10 µg/mL α-pheromone for 6 h at room temperature is shown. Blot was first blotted with anti-phosphorylated-activation-loop antibody (anti P44/42 MAPK polyclonal antibody) to detect phosphorylated forms of Cek1 (48.6 kDa), Cek1-GFP (75.7 kDa), Cek2 (43.3 kDa), and Cek2-GFP (70.5 kDa) followed by stripping and blotting with anti-α-tubulin antibody as a loading control.

At this point, there was still not conclusive evidence that the signaling cascade was fully inactivated in the *ste11Δ/Δ* mutant. To test this hypothesis, we verified whether the loss of the MEKK Ste11 affected the phosphorylation of the MAP kinases Cek1 and Cek2 using an anti-phosphorylated-activation-loop antibody. The phosphorylated forms of Cek1 (48.6 kDa) and Cek1-GFP (75.7 kDa) were detected in the wild-type strain, as well as the *cek2Δ/Δ*, GFP-tagged Cek2 and GFP-tagged Cek1 strains when exposed to pheromone treatment (Figure 6). Similarly, Cek2 (43.3 kDa) and Cek2-GFP (70.5 kDa) were also phosphorylated in response to pheromone treatment in the wild-type, GFP-tagged Cek1 and GFP-tagged Cek2 strains (Figure 6). The lack of *STE11* abrogated the transmission of the signaling to activate the MAP kinases Cek1 and Cek2 under pheromone treatment (Figure 6). The influence of Ste11 on the phosphorylation and cell localization of the MAP kinases suggest that Ste11 has not only an enzymatic activity but also a physical regulatory role in locking the MAP kinase Cek1 in the cytoplasm to make it available for phosphorylation and activation by Hst7.

### *STE11* is required for activated pheromone response in opaque cells

**Figure 7:**
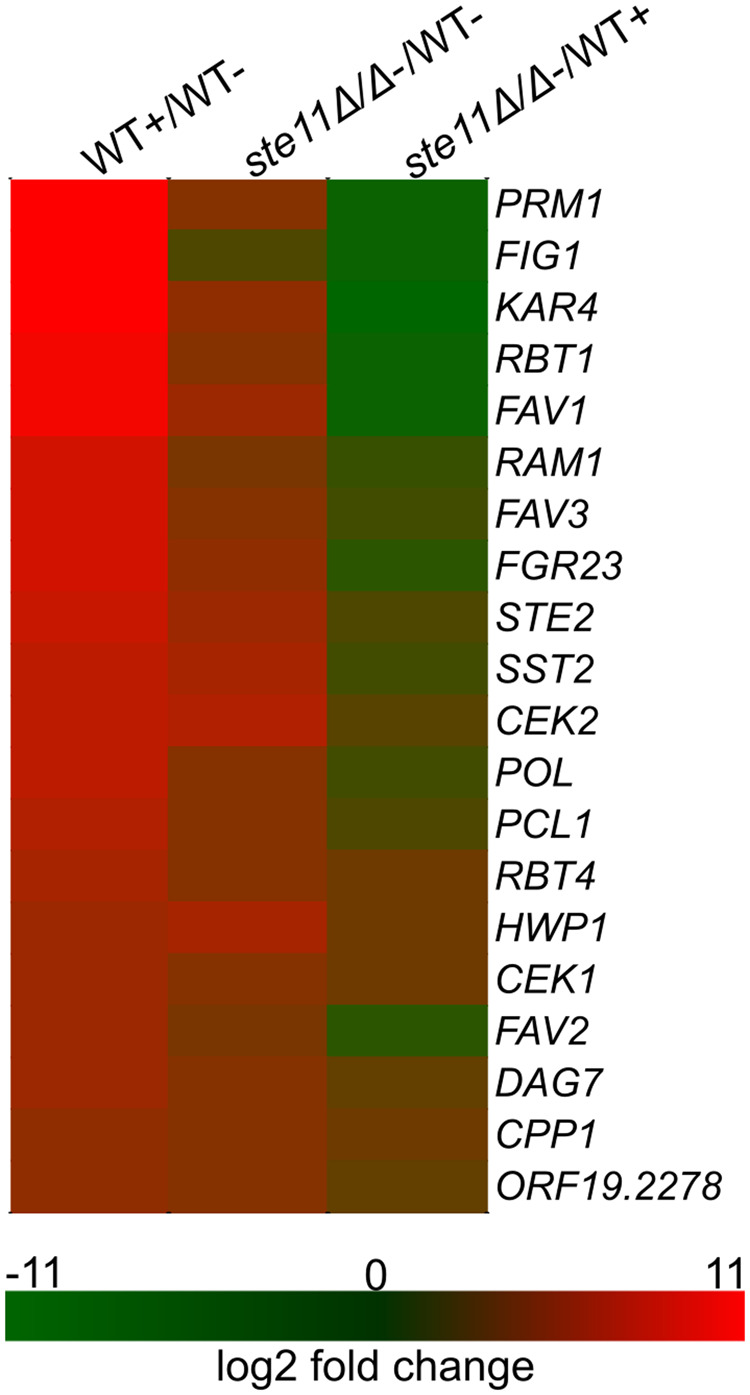
Pheromone-responsive genes were not fully activated in the absence of *STE11*. Heat map of differential expression of 21 reference pheromone-responsive genes (Zhao *et al.* 2005; Rastghalam *et al.* 2019) in opaque cells non-treated (−) or treated (+) with 10 µg/mL α-pheromone for 6 h at room temperature for the comparisons treated wild-type (WT+) *vs* non-treated wild-type (WT−), non-treated *ste11Δ/Δ vs* WT− and non-treated *ste11Δ/Δ* vs. WT+.

One of the responses to pheromone stimulation is the induction of pheromone responsive genes. As the non-stimulated *ste11Δ/Δ* mutant appeared to influence the key components Cst5 and Cek1 in spite of the absence of a true response, we assessed whether the loss of *STE11* altered transcriptional induction. The transcription profile of *ste11Δ/Δ* mutant was marked by a dramatic block in the induction of pheromone responsive genes when compared with the treated wild-type strain, while the comparison between *ste11Δ/Δ* mutant and the non-treated wild-type cells revealed an enhanced expression of some components of the pheromone pathway, specifically *STE2*, *SST2* and *CEK2* (Figure 7). As well, relative to the non-treated wild-type cells, the *ste11Δ/Δ* mutant up-regulated genes involved in RNA metabolism (Supplementary material S1). However, the expression of the components *CST5* and *CEK1* were not altered in the *ste11Δ/Δ* mutant compared with the non-treated wild-type (Supplementary material S1). Because *ste11Δ/Δ* mutant cells are sterile, defective for shmoo formation, and transcriptionally unresponsive, but can still reposition the scaffold protein Cst5 and the MAP kinase Cek1 in the absence of pheromone, these findings suggest Ste11 is a bifunctional protein with enzymatic and structural roles in the pheromone response pathway.

## Discussion

The present work proposes that the pheromone response pathway in *C. albicans* opaque cells involves complexes of proteins that regulate one another by physical interaction in both a “standby” mode in the absence of pheromone, as well as during pheromone response. Our model, building on the well-characterized *S. cerevisiae* pathway, suggests that after transmission of the signal through the pheromone receptor (Ste2 or Ste3 depending on the *MTL* locus of the strain), the activation of the heterotrimeric G protein modifies the relationship of the subunits, dissociating the Gα (Cag1) subunit from the Gβγ subunits Ste4 and Ste18. Then, the dissociated Gβγ subunit at the plasma membrane recruits the scaffold protein Cst5, which acts as a tether for the protein kinases, Ste11, Hst7 and Cek1 (Côte *et al.* 2011). The recruited Cst5-kinase complex engages with the membrane-associated Cst20 kinase, which phosphorylates and activates the MEKK Ste11, which in turn phosphorylates the MEK Hst7, which then acts on Cek1. The phosphorylated and activated Cek1 moves to the nucleus to ultimately trigger activation of Cph1, in part through the inactivation of Dig1 (Regan *et al.* 2017) resulting in the transcription of the mating-related genes. Our current observations suggest well as its enzymatic role, the MEKK Ste11 also plays a structural role in the mating process.

Scaffolds are thought to tether proteins in close proximity to ensure proper interactions and also to insulate the proteins from associations with other pathways (Elion 2001; Good *et al.* 2009). In yeast, the scaffold protein Ste5 ensures specific response to pheromone stimulus by anchoring the kinases of the MAP kinase cascade in a complex at the plasma membrane, allowing propagation of the mating signal (Elion 2001; Takahashi and Pryciak 2008; Good *et al.* 2009). Our live-cell images revealed a clear localization response of Cst5 in punctate structures at the tips of the sexual projections; however, there was no evidence of nuclear localization of Cst5-GFP in the non-treated cells in the conditions used in this study, in contrast to the behavior of Ste5 in *S. cerevisiae* (Pryciak and Huntress 1998; Mahanty *et al.* 1999).

Pryciak and Huntress (1998) demonstrated that during pheromone treatment in yeast, GFP-tagged Ste5 protein did not translocate to the cell surface in the absence of *STE4*, but the scaffold was still recruited to the cell surface in the mutants for the downstream kinases Ste20, Ste11 and Ste7. In *C. albicans* opaque cells, the mobilization of the scaffold protein seems to be controlled similarly by upstream and downstream components both in a pheromone-independent manner and in response to pheromone. Dramatic consequences of protein reorganization are seen in the *ste11Δ/Δ* mutant strain. *CST5* was not transcriptionally up-regulated relative to the untreated wild-type in the *ste11Δ/Δ* strain, and the wild-type strain exhibited no Cst5-GFP signal, but *ste11Δ/Δ* displayed a high GFP-tagged protein signal even in the absence of pheromone treatment. The appearance of a significant Cst5-GFP signal in *ste11Δ/Δ*, in the clear absence of enhanced *CST5* expression, is unexpected. One possibility is that the signal appears due to a change in the context of the protein associations or cellular localization that allows enhanced signaling in the absence of Ste11. Loss of the receptor Ste2, or the Gβ subunit Ste4 also results in the appearance of the Cst5-GFP signal dispersed in the cytoplasm of the cell, whereas this signal did not appear in the *cag1Δ/Δ* or *hst7Δ/Δ* mutant strains. The appearance of the Cst5-GFP signal in the *ste4Δ/Δ* strain, but not in the *cag1Δ/Δ* mutant, suggests these two G protein subunits have distinct relationships with Cst5, although both are positively needed for mating (Dignard *et al.* 2008). The Gα subunit seems to function only when the pathway is activated by pheromone while the Gβ subunit might play a role in regulating the scaffold protein in the standby mode as well.

These results also highlight distinct roles for the protein kinases Ste11 and Hst7, both of which are predicted to function downstream of the G protein. We observed that in the *ste11Δ/Δ* mutant pheromone treatment caused the GFP-tagged Cst5 to translocate to the plasma membrane, forming evident puncta similar to those formed in wild-type cells, but distributed in a non-oriented manner. Prior to pheromone treatment, the *ste11Δ/Δ* mutant also produced the Cst5-GFP signal at high levels, but the puncta, while present, were not as large and obvious (Figure 2, A–C). By contrast, in the *hst7Δ/Δ* mutant Cst5-GFP fluorescence was not detected in either the absence or presence of pheromone. However, double mutants for *STE11* and *HST7* exhibited a high Cst5-GFP signal independent of pheromone stimulation, which would be consistent with the loss of Ste11 allowing the appearance of Cst5-GFP regardless of the status of the downstream MAP kinase cascade. The pheromone-mediated restructuring of this signal into large, membrane associated puncta in the *ste11Δ/Δ* mutant, and to a certain extent in the *ste11Δ/Δ hst7Δ/Δ* double mutant, is consistent with a receptor-G protein mediated complex formation driven by receptor binding of the pheromone, similar to the situation in *S. cerevisiae* (Pryciak and Huntress 1998), which does not need the downstream kinases. The large pheromone-mediated puncta do not form in the *ste2Δ/Δ* and *ste4Δ/Δ* mutants, even though the Cst5-GFP signal is visible in the treated and untreated cells. Because the *C. albicans* scaffold protein is not primarily controlled in the nucleus as it is in yeast, and it takes part in a further process such a formation of sexual biofilms by *MTL*-locus homozygous white cells stimulated by pheromone (Yi *et al.* 2011), it is possible that Cst5 is tightly regulated by protein interactions in the cytoplasm to ensure proper function.

In *S. cerevisiae*, tagging of the scaffold protein Ste5 to the membrane allowed pheromone signaling in cells lacking Ste2 and the Gβγ subunits (Pryciak and Huntress 1998; Takahashi and Pryciak 2008). However, in *C. albicans* CAAX-tagged Cst5 had no impact on mating and failed to suppress any of the pheromone pathway mutants. Similarly, the strain *STE11-CAAX/STE11-CAAX* containing a membrane targeted Ste11 could not mate or form sexual projections in the absence of *CST5* though overexpression of Ste11 had been reported to partially rescue the pheromone response defect of *cst5Δ/Δ* (Yi *et al.* 2011). In *S. cerevisiae*, hyperactivated Ste11 forms can significantly bypass the absence of the scaffold protein (Stevenson *et al.* 1992; Yerko *et al.* 2013). Our findings demonstrated that tagging Cst5 and Ste11 to the plasma membrane was not enough to generate signaling to trigger pheromone responses in the absence of pheromone components due to indispensable interactions among the upstream and downstream components that appear to tightly regulate the activation of the MAP kinase cascade in *C. albicans*.

We also investigated the consequences of protein localization of the MAP kinase components of the pathway. Deletion of *STE11* significantly increased the Cek1-GFP signal cytoplasmic background and protein accumulation in the nucleus in the absence of pheromone exposure to levels higher than that for the treated wild-type strain. In contrast, it did not affect the Cek2-GFP signal, although the MEKK was still required for the pheromone-stimulated phosphorylation of both MAPKs. Recently, our group has reported that the MAP kinases Cek1 and Cek2 are both required for the mating process but play distinct roles (Rastghalam *et al.* 2019). Although Cek2 can maintain some level of mating in the absence of Cek1, its major role appears to be in adaptation to signaling (Rastghalam *et al.* 2019). Consistent with this, phosphorylation of Cek2 was undetectable early in the response (Rastghalam *et al.* 2019) but clearly evident at 6 h (Figure 6). Côte *et al.* (2011) demonstrated that Cst5 interacts with the kinases Ste11, Hst7 and Cek1, the scaffold protein Far1 and with another molecule of Cst5 independent of a vWA domain present in Ste5. They also noted direct binding between Ste11 and Hst7 through an acidic insert in the activation loop of Ste11 that is absent from ScSte11 but is closely related to the Ste7-binding loop present on Ste5, suggesting an additional role for *C. albicans* MEKK Ste11. Furthermore, yeast-two-hybrid assay revealed interactions between Ste11 and its downstream MEK Hst7 and the MAPK Cek1 (Côte *et al.* 2011). Thus, we considered that the interaction of Ste11 with Cek1 could make the MAPK available for efficient phosphorylation and activation by Hst7 in the same manner as the vWA domain of Ste5 mediates the interaction between Ste7 and Fus3 (Good *et al.* 2009). In addition, the concentration of inactivated Cek1-GFP in the nucleus in the absence of Ste11 in the untreated cells may suggest that the natural Cek1 localization is in the nucleus. Even though both MAP kinases are sensitive to pheromone stimulus, the critical interaction between Ste11 and Cek1 but not Cek2 (Côte *et al.* 2011) may contribute to a time-effective response and specific outcomes over time (Takahashi and Pryciak 2008; Conlon *et al.* 2016).

Although the deletion of Ste11 did not block the pheromone-mediated formation of cell surface puncta structures involving Cst5, signaling was still inactivated. The induction of pheromone-responsive genes was essentially blocked in the *ste11Δ/Δ* mutant in comparison with the non-treated wild-type. Intriguingly, the *ste11Δ/Δ* mutant somewhat up-regulated the pheromone pathway components Ste2 and the MAPK Cek2 despite the absence of signaling transmission and a detectable Cek2-GFP signal. These two components were also up-regulated in the absence of the phosphatase Cpp1 and the MAP kinase Cek2 (Rastghalam *et al.* 2019), suggesting Ste11 may be also a regulatory component of the pathway. Overall, genes annotated for RNA metabolism made up the majority of genes up-regulated in *ste11Δ/Δ* versus non-treated wild-type (Supplementary material S1) as well as in the inactivated Cek2 mutant compared with the non-treated wild-type in the absence of pheromone (Supplementary material S1) (Rastghalam *et al.* 2019). In spite of there being no Cek2-GFP signal in the *ste11Δ/Δ* mutant, these observations suggest a linkage for Ste11 and Cek2 in the regulation of the pathway.

CaSte11 shares 59% sequence identity with ScSte11. The MEKK Ste11 has two N-terminal regulatory domains in both organisms, SAM and RBL, which in yeast interact with the adaptor protein Ste50 and the scaffold protein Ste5, respectively, to ensure the signal is properly related to the environmental cues (Yerko *et al.* 2013). In *C. albicans*, the C-terminal kinase domain of Ste11 is responsible for phosphorylation and docking of Hst7 (Côte *et al.* 2011). Furthermore, we demonstrated that Ste11 seems to be involved in restricting the MAPK Cek1 in the cytoplasm to make it available for phosphorylation and activation by Hst7. Indeed, Ste11 may form a stable complex with Cst5 in order to hold the MAPK proteins in proximity to ensure that the signal is properly transmitted. The activation of the MAPK cascade as a result of the association of the scaffold protein with the MAPK proteins would be stabilized by Ste11 as well. After phosphorylation by Hst7, Cek1 would change affinity to Ste11 being released and moving to the nucleus to act on the transcription factor Cph1 inducing the pheromone responses. The functional flexibility of CaSte11 has already been demonstrated to impact on the upstream step of mating process allowing a hyperactive CaSte11 (lacking the regulatory domain) to induce white-opaque switching independent of Cst5 and under influence of an unknown stimulus through activation of Cek1 and downstream transcription factor Cph1 (Ramírez-Zavala *et al.* 2013). Despite of a simplified scaffold protein, this human fungal pathogen was able to modulate extant proteins in order to rearrange the protein-protein interactions to achieve proper signaling transmission.
